# Depressed “ping pong” skull fractures in the newborns: A cohort study

**DOI:** 10.1002/hsr2.2283

**Published:** 2024-09-25

**Authors:** Suhair M. A. Qudsieh, Mohammed M. Al Barbarawi, Omar F. Altal, Ala M. Al Barbarawi, Raed M. Al‐Zoubi, Mazhar S. Al Zoubi

**Affiliations:** ^1^ Department of Clinical Sciences, Division of Obstetrics and Gynecology, Faculty of Medicine Yarmouk University Irbid Jordan; ^2^ Department of Neurosurgery, Faculty of Medicine Jordan University of Science and Technology Irbid Jordan; ^3^ Department of Obstetrics and Gynecology, Faculty of Medicine Jordan University of Science and Technology Irbid Jordan; ^4^ Faculty of Medicine Jordan University of Science and Technology Irbid Jordan; ^5^ Surgical Research Section, Department of Surgery Hamad Medical Corporation Doha Qatar; ^6^ Department of Biomedical Sciences, QU‐Health, College of Health Sciences Qatar University Doha Qatar; ^7^ Department of Basic Medical Sciences, Faculty of Medicine Yarmouk University Irbid Jordan

**Keywords:** depressed skull fracture, difficult delivery, instrumental delivery, neonate, newborn, ping pong fracture, skull fracture elevation

## Abstract

**Background and Aims:**

A ping pong fracture is a rare depressed skull fracture (DSF) observed in infants. It occurs due to the inward buckling of the calvarium, creating a cup‐like shape. Trauma during childbirth, particularly from instrumental delivery or the application of pressure by physicians or midwives during challenging deliveries, is the primary cause. This study aimed to investigate the epidemiologic characteristics associated with DSF in newborns and to identify the main factors related to its incidence and the type of hematoma involved.

**Methods:**

This is a retrospective case‐control analysis of all newborns delivered with DSF at King Abdulla University Hospital in Jordan between January 2008 and December 2020. The medical records were reviewed, and clinical data were collected and analyzed.

**Results:**

Out of 42,955 live births delivered at King Abdulla University Hospital, 13 cases of DSF were observed, giving an incidence of 3.0 in 10,000 live births. All cases were delivered at full term. Of the 13 cases, nine cases were associated with the use of instrumental delivery. Seven of those nine cases were delivered vaginally, while the other two cases required cesarean section following unsuccessful instrumental delivery. Four cases were spontaneous, with no history of trauma or instrument use, and delivered by cesarean section. Only 3 of the 13 cases required neurosurgical elevation of DSF. The outcome was excellent in all cases, both cosmetically and neurologically.

**Conclusion:**

Ping‐pong skull fractures are seen in newborns infrequently in the Jordanian population, with an incidence of 0.03%. Most of the cases have resulted from difficult deliveries though spontaneous fractures can be encountered rarely. The treatment is usually conservative with spontaneous resolution. The overall prognosis is excellent both neurologically and cosmically.

## 1 INTRODUCTION

Neonatal depressed skull fracture (DSF), also known as Ping pong skull fracture, denotes a DSF of the infant skull caused by the inner buckling of the calvarium without loss of bone continuity.^1^ These are rare in modern perinatal medicine due to advances in training and techniques, with an incidence of 1−2.5 in 10,000 births.^2^, ^3^, ^4^ A retrospective case‐control study from France of 1,994,250 deliveries over 10 years reported 75 cases of DSF with an incidence of 1 in 26,000 deliveries,^5^ while a study from India reported four instances of DSF in 34,946 deliveries (i.e., 1.1 in 10,000 live deliveries.^6^ The occurrence of these fractures has been related to prolonged labor, fetal head positioning during labor, or inexperienced use of instrumental deliveries.^7^ Furthermore, DSF might occur spontaneously without instrumentation or a history of trauma during pregnancy or delivery.^1^, ^3^ It occurs mostly in parietal‐temporal skull bones as they are soft.

The management options include surgical or nonsurgical elevation techniques and conservative treatment with spontaneous resolution.^8^ This depends on the severity of the fracture and the association with intracerebral injury. In certain instances, opting for conservative management, particularly when the neonate does not exhibit abnormal neurological signs, has led to spontaneous resolution.^9^ However, if the fracture is large with a depth of more than 2 cm or associated with mechanical compression on the brain, reduction by neurosurgical elevation should be considered.^10^ Most untreated ping‐pong fractures resolve spontaneously within 6 months.^1^, ^3^, ^11^, ^12^ The literature showed that spontaneous DSF has a good prognosis, although some research reported that 4% of traumatic DSF have long‐term consequences resulting in neurological disabilities.^1^, ^5^, ^8^

In this study, we retrospectively reviewed all DSF cases reported in the last 12 years from a tertiary referral center in Jordan. We explored the common epidemiologic characteristics related to DSF patients and investigated the main clinical factors associated with the trauma and hematoma types in DSF.

## 2 METHODS

This retrospective study was approved by the Institutional Research Board (IRB) at Jordan University of Science and Technology (JUST) and King Abdullah University Hospital (KAUH) in Jordan. The ethical approval (IRB) number is 56‐2022. The study group comprised all newborn patients with ping pong fractures treated at our institution between January 2008 and December 2020. All newborns born with calvarium depression were included.

The study group consisted of 13 newborns out of 42,955 live births diagnosed with DSF immediately after vaginal delivery or cesarean section. Cases were classified as spontaneous if delivery was not associated with instrument use to assist delivery (*n* =  4 cases). On the other hand, cases in which forceps or vacuum cups were used to assist delivery either successfully or unsuccessfully, were classified as traumatic “instrument‐associated” (*n* =  9 cases).

**Figure 1:**
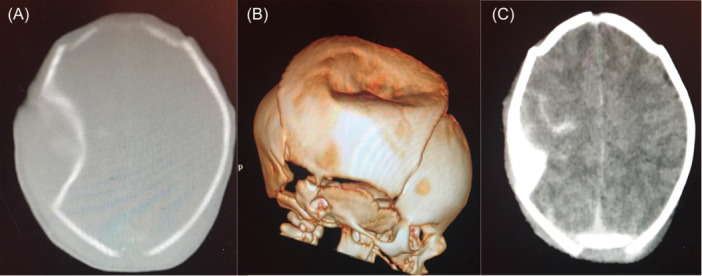
Preoperative (A) axial Computed tomography (CT) scan bone window, (B) 3D CT scan, and (C) plain CT scan. Images show a large frontoparietal ping pong fracture of 7 × 5 × 2 cm in diameter, compressing the underlying brain structure and associated with traumatic subarachnoid hemorrhage.

All patients underwent full clinical and neurological assessment by a neonatologist and neurosurgeon, and none of them demonstrated focal neurological deficits. All cases with skull depression underwent a CT scan with bone window and 3D reconstruction view. Ten cases had mild to moderate DSF and were treated conservatively, while only three cases had large, depressed fractures with underlying cerebral compression and a deformed skull that required surgical elevation for release of the mechanical cerebral compression and cosmetic reasons (Figure 1). The infants were followed‐up in the outpatient department at 2 weeks, 6 weeks, 3 months, 6 months, and 1 year.

### 2.1 Surgical technique

**Figure 2:**
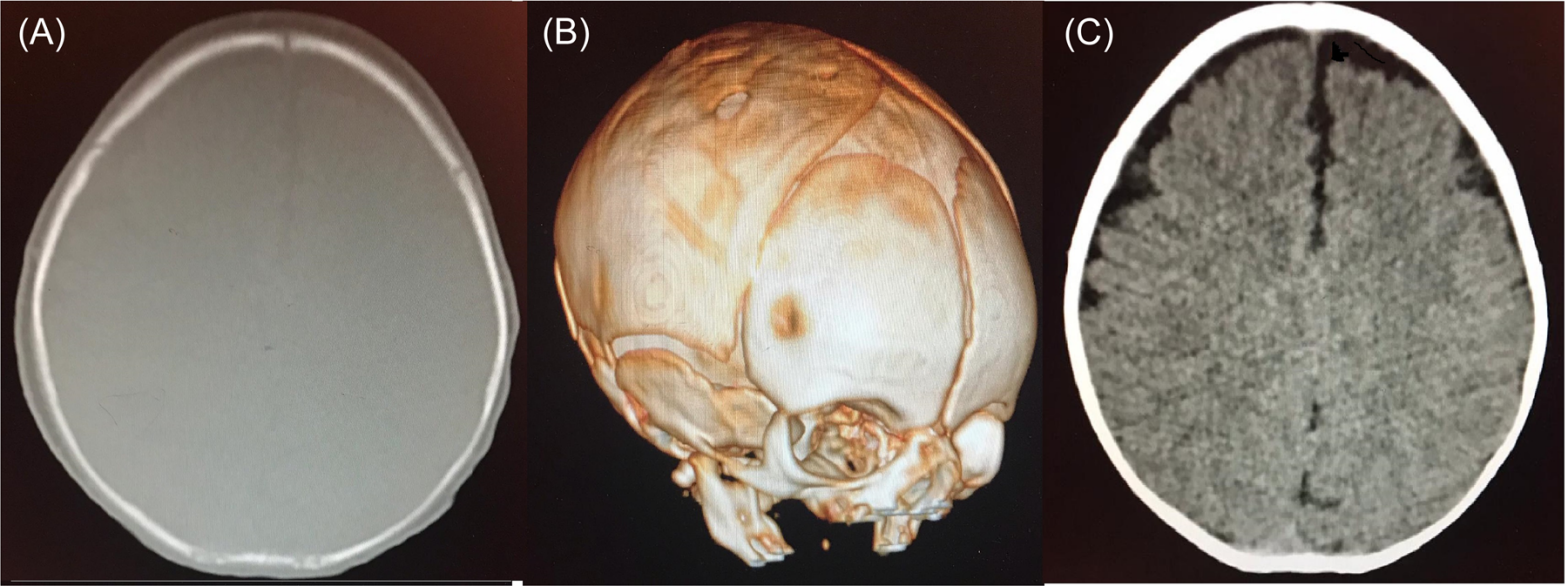
Postoperative (A) axial Computed tomography (CT) scan bone window, (B) 3D CT scan, and (C) plain CT scan images show remodeling of the calvarium with normal skull reconstruction and underlying brain.

Surgical intervention was conducted within 48 h of delivery for three cases. Under general anesthesia, with endotracheal intubation, the patient was positioned supine with the head up and tilted to the opposite side. A small skin incision of 1−2 cm in length was made at the posterior rim of the bone depression. Subsequently, a burr hole was created, followed by a gentle introduction of the Watson chain tool between the dura and bone to elevate the depression gradually and smoothly. The skin was then closed in layers, and dressing was applied. The three patients had a full and smooth recovery with an uneventful postoperative course. Blood loss was negligible. The postoperative CT scan with a 3D view was performed on postoperative day one with excellent results (Figure 2).

### 2.2 Statistical analysis

The factors that were investigated concerning DSF were described using frequency distribution for categorical variables and mean ± standard deviation for continuous variables. Pearson's chi‐square (*χ* ^2^) test was used to analyze the associations between categorical variables, and Student's *t*‐test was used for continuous variables. Statistical significance was set at *p* < 0.05. If a substantial association was found between categorical variables, a post hoc residual analysis was conducted to determine the exact significance of the contingency tables.

## 3 RESULTS

The incidence of DSF at our institution is 3.0 in 10,000 live births. The characteristics of the patients are presented in Table 1. All cases were delivered at full term. The average birth weight was 3.4 kg, ranging between 2.9 and 3.9 kg. The majority of cases were males (76.9%). Nine cases (69.5%) had traumatic fracture types and were associated with the use of assisted delivery instruments. Four cases (30.8%) had a spontaneous fracture type as no instruments were used to assist delivery. Six cases (46.2%) were delivered by cesarean section where the head was deeply impacted in the pelvis and delivered with difficulty, two of them followed unsuccessful instrumental delivery. Eight cases (61.5%) were associated with scalp hematoma, while five cases (38.5%) were associated with intracranial hemorrhage. All skull fractures involved the parietal bone.

**Table 1:** Characteristics and clinical presentations of depressed skull fracture patients.

	Number	Percent (%)
Characteristics	**Mean ± SD**	
Total number of cases	13	100
Sex		
▪ Male	10	76.9
▪ Female	3	23.1
Weight (kg)	3.4 ± 0.4	
Order of current delivery		
▪ 1st	5	38.5
▪ 2nd	2	15.4
▪ 3rd	2	15.4
▪ 4th and more	4	30.8
Head position		
▪ Occipito‐anterior	6	46.2
▪ Occipito‐posterior	5	38.5
▪ Occipito‐transverse	2	15.4
Fracture type		
▪ Trauma	9	69.2
▪ Spontaneous	4	30.8
Fracture side		
▪ Right	6	46.2
▪ Left	7	53.8
Use of assisted instruments	9	69.2
Required cesarean section	6	46.2
Associated hematoma		
▪ Scalp hematoma	8	61.5
▪ Intracranial hematoma	5	38.5
Facial palsy	2	15.4
Brachial plexus injury	0	0.0
Eye injury	0	0.0

Factors associated with the type of DSF (traumatic vs. spontaneous) are summarized in Table 2. There was a significant (*p* < 0.05) association between the head position and the type of fracture, as newborns with occipital‐anterior head position were more exposed to traumatic skull fracture.

**Table 2:** Factors associated with the fracture type in depressed skull fracture patients.

Characteristics	Traumatic fracture *N* (%)	Spontaneous fracture *N* (%)	*p* Value
Total number of cases	9 (69.2)	4 (30.8)	‐
Sex			NS
▪ Male	7 (77.8)	3 (75.0)	
▪ Female	2 (22.2)	1 (25.0)	
Weight (kg), mean ± SD	3.5 ± 0.4	3.3 ± 0.4	NS
Order of current delivery			NS
▪ 1st	3 (33.3)	2 (50.0)	
▪ 2nd	1 (11.1)	1 (25.0)	
▪ 3rd	1 (11.1)	1 (25.0)	
▪ 4th and more	4 (44.4)	0 (0.0)	
Head position			<0.05
▪ Occipito‐anterior	6 (66.7)^**↑**^	0 (0.0)	
▪ Occipito‐posterior	2 (22.2)	3 (75.0)	
▪ Occipito‐transverse	1 (11.1)	1 (25.0)	
Fracture side			NS
▪ Right	4 (44.4)	2 (50.0)	
▪ Left	5 (55.6)	2 (50.0)	
Required cesarean section	2 (22.2)	4 (100)	NS
Associated hematoma			NS
▪ Scalp hematoma	6 (66.7)	2 (50.0)	
▪ Intracranial hematoma	3 (33.3)	2 (50.0)	

Abbreviations: N, number; NS, not significant; P, probability; SD, standard deviation.

Factors associated with the hematoma type (scalp vs. intracranial) are summarized in Table 3. There was a significant (*p* < 0.05) association between cesarean delivery and the type of associated hematoma, as newborns who underwent cesarean section had more incidence of intracranial hemorrhage.

**Table 3:** Factors associated with hematoma type in depressed skull fracture patients.

**Characteristics**	**SCALP hematoma *N* (%)**	**Intracranial hematoma *N* (%)**	***p* Value**
Total number of cases	8 (61.5)	5 (38.5)	‐
Sex			NS
▪ Male	6 (75.0)	4 (80.0)	
▪ Female	2 (25.0)	1 (20.0)	
Weight (kg), mean ± SD	3.4 ± 0.5	3.4 ± 0.3	NS
Order of current delivery			NS
▪ 1st	3 (37.5)	2 (40.0)	
▪ 2nd	1 (12.5)	1 (20.0)	
▪ 3rd	1 (12.5)	1 (20.0)	
▪ 4th and more	3 (37.5)	1 (20.0)	
Head position			NS
▪ Occipito‐anterior	4 (50.0)	2 (40.0)	
▪ Occipito‐posterior	3 (37.5)	2 (40.0)	
▪ Occipito‐transverse	1 (12.5)	1 (20.0)	
Fracture type			NS
▪ Traumatic	6 (75.0)	3 (60.0)	
▪ Spontaneous	2 (25.0)	2 (40.0)	
Fracture side			NS
▪ Right	5 (62.5)	1 (20.0)	
▪ Left	3 (37.5)	4 (80.0)	
Required cesarean section			<0.05
▪ Yes	2 (25.0)	4 (80.0)^↑^	
▪ No	6 (75.0)	1 (20.0)	

Abbreviations: N, number; NS, not significant; P, probability; SD, standard deviation.

A conservative approach was used in the majority of the cases (*n* = 10), while only three cases required surgical elevation for release of mechanical cerebral compression and cosmetic reasons. The three patients who had surgery underwent a repeat CT scan with bone window the next day postoperation and showed satisfactory outcomes. The patients who were treated conservatively had a repeat CT scan with bone window at 2 weeks of age and then at 6 weeks of age, if the skull went back to normal, no further CT scan imaging is required. The infants were followed‐up in the outpatient department at 2 weeks, 6 weeks, 3 months, 6 months, and 1 year. On the short and long‐term follow‐up, all cases showed full recovery within 6 months. The outcome was excellent for all infants, whether treated conservatively or surgically.

## 4 DISCUSSION

Bone injuries during delivery have been reported as 1 in 1000 live births. The clavicle is the most common bone fracture representing half of the cases, while DSF accounts for 11%.^4^ DSF in newborn infants at birth are rare, with an incidence ranging between 1 and 2.5 per 10,000 births.^7^, ^11^ In our study, the observed incidence is 3 in 10,000 live births, which is higher than other reports. This disparity is likely attributed to the fact that our institution functions as a referral and university hospital, providing medical care to a substantial volume of patients, including those deemed high‐risk. On the other hand, a retrospective study from France on obstetric DSF over 10 years, showed an incidence of 1: 26,000 live births. Out of 68 cases, 50 (73.5%) were instrumental, and 18 (26.5%) were spontaneous (i.e., no instruments were used). There were 34 (68%) newborns delivered vaginally from the instrumental group, while eight (44.4%) cases were delivered vaginally from the spontaneous group.^5^

Nine cases in our study can be described as traumatic skull fractures as they resulted from trauma during delivery, most probably due to the use of instruments. The instrumental delivery caused 9 out of 13 (69.2%) cases of DSF, which is comparable to other studies.^5^ In the literature, most cases resulting from perinatal trauma were associated with the use of obstetric instruments or applied obstetric maneuvers to assist in the delivery of the head during difficult labor.^3^, ^7^, ^13^ However, spontaneous (atraumatic) DSF during vaginal delivery may result from pressure on the fetal head from the mother's pelvic bony prominences like ischial tuberosity, ischial spines, sacral promontory, and pubic bones. The prolonged pressure on the calvarium can result in a localized depression of the skull, giving the appearance of a ping‐pong ball due to inward buckling of the calvaria even in the absence of instrumental use.^3^, ^11^ Both fracture types (traumatic or spontaneous) have medical and legal implications for the physician. If the DSF is suspected, then a head CT scan is recommended to assess the degree of skull deformity and the presence of brain injury.^3^, ^11^

The management options include conservative and surgical treatment, the choice depends on the severity of the fracture and the presence of intracerebral injury. Surgical treatment is indicated if the defect is large or if there is cerebral injury or compression.^11^, ^14^ However, most cases are managed conservatively. There are many treatment options described in the literature. These include watchful waiting, nonsurgical reduction using suction from a vacuum extractor or breast pump, digital pressure on the edges of the depression and surgical treatment using the standard method of DSF.^3^, ^8^

Compared to other studies, the outcome for our patients was excellent. All cases had normal skull growth and development. There was no difference in the outcome between affected neonates born by vaginal delivery or cesarean section.

## 5 CONCLUSION

DSF or ping pong fractures are seen infrequently in newborns, with a notable incidence of 3 per 10,000 live births. Most of these cases have resulted from difficult delivery and are related to the inexperienced use of obstetric instruments. However, spontaneous fractures can also be encountered. Treatment is usually conservative, but surgery may be required with simple surgical elevation of the depressed bone in certain cases.

## AUTHOR CONTRIBUTIONS

**Mohammed M. Al Barbarawi**: Data curation; investigation; formal analysis; methodology; writing—review and editing. **Omar F. Altal**: Methodology; formal analysis; data curation; writing—review and editing; investigation. **Ala M. Al Barbarawi**: Methodology; investigation; formal analysis; data curation. **Raed M. Al‐Zoubi**: Writing—review and editing; writing—original draft; investigation; data curation; formal analysis; methodology. **Mazhar S. Al Zoubi**: Methodology; data curation; formal analysis; investigation; writing—review and editing. All authors have read and approved the final version of the manuscript.

## CONFLICT OF INTEREST STATEMENT

The authors declare no conflict of interest.

## ETHICS STATEMENT

This study was approved by the Institutional Research Board (IRB # 56‐2022) at Jordan University of Science and Technology (JUST) and King Abdullah University Hospital (KAUH).

## TRANSPARENCY STATEMENT

The lead author Suhair M. A. Qudsieh, Raed M. Al‐Zoubi affirms that this manuscript is an honest, accurate, and transparent account of the study being reported; that no important aspects of the study have been omitted; and that any discrepancies from the study as planned (and, if relevant, registered) have been explained.

## Data Availability

The data that support the findings of this study are available from the corresponding author upon reasonable request. The corresponding author had full access to all of the data in this study and took complete responsibility for the integrity of the data and the accuracy of the data analysis.
